# Severity and progression of structural hand OA is not associated with progression of structural knee OA: The IMI-APPROACH cohort

**DOI:** 10.1016/j.ocarto.2024.100487

**Published:** 2024-05-14

**Authors:** Sietse E.S. Terpstra, Lotte A. van de Stadt, Francis Berenbaum, Francisco J. Blanco, Ida K. Haugen, Simon C. Mastbergen, Harrie Weinans, Mylène P. Jansen, Frits R. Rosendaal, Margreet Kloppenburg

**Affiliations:** a Department of Rheumatology, Leiden University Medical Center, the Netherlands; b Department of Clinical Epidemiology, Leiden University Medical Center, Leiden, the Netherlands; c Department of Rheumatology, Amsterdam Rheumatology and Immunology Center|Reade, Amsterdam, the Netherlands; d Sorbonne University, Inserm, APHP Hôpital Saint-Antoine, Paris, France; e Grupo de Investigación de Reumatología (GIR), INIBIC – Complejo Hospitalario Universitario de A Coruña, SERGAS. Centro de Investigación CICA, Departamento de Fisioterapia y Medicina, Universidad de A Coruña, A Coruña, Spain; f Center for Treatment of Rheumatic and Musculoskeletal Diseases (REMEDY), Diakonhjemmet Hospital, Oslo, Norway; g Department of Rheumatology & Clinical Immunology, University Medical Center Utrecht, Utrecht University, Utrecht, the Netherlands; h Department of Orthopedics, University Medical Center Utrecht, Utrecht University, Utrecht, the Netherlands

**Keywords:** Osteoarthritis, Structural, Knee, Hand

## Abstract

**Objective:**

To investigate whether structural hand OA or its progression is associated with structural knee OA progression after two years in a population with symptomatic knee OA.

**Methods:**

We used baseline and two-year follow-up data from the IMI-APPROACH cohort. Symptomatic hand and knee OA were defined using ACR criteria. Radiographs of hands and knees were scored semi-quantitatively for osteophytes and joint space narrowing (JSN) following the OARSI atlas, and Kellgren-Lawrence (KL) scale. Knee images were also scored quantitatively with the Knee Image Digital Analysis (KIDA). Progression was defined as change above the minimal detectable change on patient level, except for KIDA (most affected knee compartment level). With logistic regression analyses the severity or progression of hand OA was associated with knee OA progression.

**Results:**

In 221 participants (mean age 66, 77% women, mean BMI 27.7, 19% hand OA), OA progression occurred in 18%–28%, and 9%–38% in hands and knees respectively, depending on features. Baseline structural hand OA features were not significantly associated with knee OA progression, except for hand osteophytes with KIDA osteophytes progression (odds ratio (OR) 1.03, 95% confidence interval (CI) 1.01–1.06). Progression of structural hand OA features was not significantly associated with knee OA progression, except for hand osteophyte or JSN progression, which was significantly associated with knee osteophyte progression (OR 0.44, 95%CI 0.22–0.84 and OR 0.43, 95%CI 0.18–0.94, respectively), and hand osteophyte progression for knee JSN (OR 2.51, 95%CI 1.15–5.48).

**Conclusions:**

In patients with symptomatic knee OA, no consistent associations between baseline structural hand OA or hand OA progression and knee OA progression were shown.

## 1 Introduction

Osteoarthritis (OA) affects almost 600 million persons worldwide in 2020 and is expected to keep rising in prevalence [1]. It is characterised by degradation of cartilage and abnormalities in subchondral bone and synovium, leading to pain and disability [2]. OA is a heterogeneous disorder, multiple clinical phenotypes are seen, such as hand OA, knee OA, hip OA, spine OA, or OA in multiple joint groups at once (“generalized OA”) [[3], [4], [5]]. As systemic factors (such as ageing, genetics, obesity and female sex) are frequent risk factors for OA, generalized OA is prevalent [6]. The disease course of OA varies between patients, as some patients experience rapid progression over time while others remain relatively stable.

One of the most prevalent clinical phenotypes of OA is knee OA [1,7], which is the most important cause for total knee replacement [8]. Knowledge of predictors of structural knee OA progression is vital for providing adequate prognosis and therapy to patients with knee OA. However, evidence for such predictors is inconclusive [9]. Structural hand OA severity on radiographs might be a potent predictor with high feasibility and low costs. Previous studies found an association between presence and progression of structural hand OA abnormalities and presence and development of structural knee OA [[10], [11], [12]]. However, another study found no consistent association between knee OA progression and hand OA progression [13]. To our knowledge, the association between structural hand and knee OA has not been investigated yet in a study population selected on high likeliness of structural knee OA progression. Doing so might provide different results than previous similar studies, as OA generally progresses slowly [14].

Therefore, we aimed to investigate the association of the severity of structural hand OA and the progression of structural hand OA with structural knee OA progression over two years of follow-up in the Applied Public-Private Research enabling OA Clinical Headway (IMI-APPROACH) cohort. This cohort is selected on expected progression of knee OA pain and structural damage [15]. We hypothesized an association of hand OA severity and progression, with knee OA progression.

## 2 Materials and methods

### 2.1 Study design

The IMI-APPROACH cohort is an European, five-center, 2-year prospective follow-up cohort study (registered under trial number NCT03883568). Participant selection and study design have been described in detail elsewhere [16,17]. In short, patients were (pre-)selected from five existing European observational cohorts using machine learning models trained to increase the inclusion of patients with a high likelihood of structural progression (assessed by “minimum joint space width”) or pain progression (assessed by “KOOS pain” scale). Participating centers included: University Medical Center Utrecht (UMCU), Utrecht, The Netherlands- Leiden University Medical Center, Leiden, The Netherlands- Diakonhjemmet Hospital, Oslo, Norway- Sorbonne University APHP Saint-Antoine hospital, Paris, France- Complexo Hospitalario Universitario de A Coruña, A Coruña, Spain. The current study describes longitudinal analyses of the data from baseline to year two. Ethical approval was obtained locally in the involved centers. All participants provided written informed consent.

### 2.2 Patient selection

During the screening visit inclusion and exclusion criteria were verified, and parameters were collected that were subsequently used in the machine learning algorithm for the final participant selection based on the highest likelihood of knee OA pain progression and/or joint space narrowing (JSN) of the knee. For inclusion, patients needed to fulfil the American College of Rheumatology clinical classification criteria for knee OA on at least one knee (index knee) [18], were able to walk unassisted and needed to be capable of understanding the study protocol. If both knees fulfilled the criteria, the most painful knee was selected as the index knee. If equal, the right one was selected as the index knee. Patients were excluded for example if they had recent surgery or had planned surgery of the index knee during follow-up, in case of secondary knee OA, alternative causes of joint pain or if a generalized pain syndrome was present. Full inclusion and exclusion criteria were published previously [17]. For this study, we included participants with available hand and knee radiographs at baseline and year two.

### 2.3 Questionnaires and clinical assessment

At baseline, general patient characteristics were collected, including age, sex, comorbidities (Charlson index [19]) and measured body weight and height for calculation of the body mass index (kg/m2).

Patients filled in the Functional Index for Hand OsteoArthritis, consisting of 10 items, completed on a 4-point Likert scale. The total score ranges from zero (“no functional impairment”), to 30 (“maximal impairment”) [20]. The Numeric Rating Scale (NRS) for pain was acquired regarding pain in the past week for each hand separately, ranging from 0 (no pain) to 10 (maximum pain) [21]. A NRS hand pain summated score for both hands was calculated, ranging from 0 to 20. Bony and soft tissue swellings and deformities of distal interphalangeal, proximal interphalangeal, metacarpophalangeal (MCP), carpometacarpal (CMC) joints were assessed. Symptomatic OA of the hands was defined according to the original American College of Rheumatology classification criteria [22], based on among others pain, aching or stiffness on most days of the last month, and physical examination.

For the knees, participants completed standardized questionnaires including an NRS scale for assessment of knee pain [21], and the Knee injury and Osteoarthritis Outcome Score (KOOS) for knee OA pain, stiffness and function [23]. From the KOOS, the pain (9 items) and function in activities of daily living (17 items) subscales were used. Items were scored on a 5-point Likert scale. Subscale scores were calculated according to the KOOS user's guide as the sum of the items included, and subsequently transformed to a 0–100 scale, with zero representing extreme knee problems and 100 representing no knee problems [24]. Both knees were assessed for palpable warmth, effusion (positive patellar tap), passive ranges of flexion and extension, grinding in the patellofemoral joint, and knee alignment (presence of valgus or varus).

### 2.4 Radiographic assessment

Bilateral radiographs of the hands and knees were obtained at baseline and two years using the same standard operating procedure in all centers, and were scored in known time order by one trained reader (LS), blinded for participant characteristics. Joints were scored according to the Osteoarthritis Research Society International (OARSI) atlas. Concerning osteophytes and JSN of the hands, bilateral DIPs, PIPs and CMC1s were scored on a 0–3 scale, and the IPJs and STTs joints on a 0–1 scale (summated score range for both hands: 0–58). For the knees, medial and lateral femoral and tibial osteophytes were scored on a 0–3 scale (summated score 0–24), and medial and lateral tibiofemoral JSN was scored on a 0–3 scale (summated knee score 0–12) [25]. The hands and knees were also scored using the Kellgren and Lawrence (KL) scale for both knees and both hands (DIPJs, PIPJs, IPJs, MCPJs and CMC1s (range 0–4 for each joint, summated knee score 0–8, summated hand score 0–120) [26]. The intra-class correlation coefficient (ICC) was calculated on a random sample of 10% of radiographs of both knees and both hands to evaluate intra-reader reliability. The ICC for all scores of the knees (KL, osteophyte and JSN) exceeded 0.91, and for the hands all ICC scores exceeded 0.93.

Osteophytes and joint space width (JSW) were also assessed with a quantitative method. The Knee Images Digital Analysis (KIDA) was used to semi-automatically quantify mean JSW of the medial and lateral knee component, and the osteophyte area (mm^2^) of the medial and lateral tibia and femur on the radiographs [27,28]. The “most affected compartment” of the index knee was assessed for progression larger than the minimal detectable change. This compartment was determined by two readers in consensus by investigating the characteristics used in OA scoring systems (JSW, osteophytes, sclerosis) using radiographs at year two, and could be either the lateral or medial compartment.

### 2.5 Definitions of severity and progression

Structural hand OA severity was defined by the summated score for each of the different features (osteophytes, JSN, and KL). Progression of individual features based on the OARSI atlas and global OA based on the KL scale was defined as a difference between baseline and year two larger than the minimal detectable change for the concerning score (the smallest difference between two independently obtained measures that can be interpreted as ‘‘real’’, as it is greater than the measurement error) (based on repeated scoring of 30 pairs of radiographs) [29]. For the KIDA outcomes, a minimal detectable change was determined previously using similar knee radiographs and the same observer [27]. Patients that had likely OA-related surgery before inclusion received a maximum score for the concerning joint at both baseline and year two. Three patients had a knee replacement during follow-up (of the index knee) and were therefore counted as progressors.

### 2.6 Statistical analyses

Logistic regression analyses were performed, and odds ratios with 95% confidence intervals (CIs) were calculated. In addition, relative risks with 95% CIs were calculated for the association of 1) structural hand OA severity at baseline (independent variable) with knee OA progression (dependent variable) and 2) structural hand OA progression (independent variable) with knee OA progression (dependent variable). As a sensitivity analysis, we repeated all logistic regression analyses excluding participants that had a knee replacement during follow-up (n ​= ​3), or had hand surgery likely related to OA before or during follow-up (n ​= ​6) instead of assigning these a maximum score for the concerning joint. We repeated all analyses concerning knee osteophytes and JSN progression, assessed with the KIDA software program instead of the OARSI atlas. In these analyses, as dependent variable knee OA progression of the most affected compartment of the index knee was assessed only, in alignment with earlier analyses in the IMI-APPROACH cohort [15]. All logistic regression analyses were performed crude and adjusted for potential confounders (age, sex and BMI). These specific potential confounders were adjusted for, as in previous literature these variables were associated both with hand OA progression and knee OA progression.

We compared the baseline characteristics of patients with versus without progression of individual OA features by the OARSI atlas in the knees (chi-squared test for dichotomous variables, *t*-test for normally distributed variables and Mann-Whitney *U* test for comparing non-normally distributed non-dichotomous variables) [30]. Similarly, we also compared patients with complete data at year two versus those lost to follow-up. We also assessed the agreement of knee OA progression according to the OARSI atlas and knee OA progression according to the KIDA. R Studio Version April 1, 1717 was used for all analyses.

### 2.7 Patient and public involvement

A patient council has been set up and has contributed to the design of the clinical study and helped shape the project to ensure consideration of the interests of study participants. The patient council has been in close contact with the study researchers throughout the project [31].

## 3 Results

### 3.1 Study population

The IMI-APPROACH cohort consists of 297 participants. Due to exclusion of patients with missing radiographic data at baseline (n ​= ​18), missing radiographic data at year two (n ​= ​25) and loss to follow-up (n ​= ​33), the population of the current analysis consisted of 221 patients. Baseline characteristics of the present study cohort are shown in Table 1. We compared all patient characteristics of Table 1 between patients included (n ​= ​221) versus excluded (n ​= ​76) (data not shown), and found a statistically significant difference only for KOOS function (median 32 (IQR 18; 47) versus 38 (25; 49), mean difference 5 (95% CI: 0–10))s. Six patients had hand surgery likely related to OA (trapeziectomy or arthrodesis) before inclusion and therefore received a maximum score for the concerning joint at both baseline and year two. Three patients had a knee replacement during follow-up (of the index knee) and were therefore counted as progressors. The number of patients with missing data for any variable did not exceed 5%.

**Table 1:** Baseline participant characteristics of the study population.

	Patients included in analyses (n ​= ​221)
**General participant characteristics**	
Age (years) (mean, SD)	66.4 (7.1)
Sex, women	170 (77%)
Body Mass Index (kg/m^2^) (mean, SD)	27.7 (5.0)
Ethnicity (white)	214 (97%)
Any comorbidity	133 (60%)
**Hand OA characteristics**	
Symptomatic hand OAˆ	41 (19%)
FIHOA score (0–30) (median, IQR)	3 (0–9)
NRS hand pain, both hands (0–10) (median, IQR)	3.0 (1–6)
Structural hand OA (KL for any hand joint >1)	154 (69%)
**Knee OA characteristics**	
Symptomatic knee OAˆ	221 (100%)
KOOS pain (0–100) (both knees, median, IQR)	30 (15–45)
KOOS ADL function (0–100) (both knees, median, IQR)	32 (18–47)
NRS knee pain, both knees (0–10) (mean, SD)	3.5 (1.5–5.5)
Structural knee OA (KL for any knee >1)	127 (57%)
Most affected compartment: medial (index knee)	185 (88%)
Most affected compartment: lateral (index knee)	35 (12%)

Numbers represent n (%) unless specified otherwise. Abbreviations: ADL = activities of daily living, OA = osteoarthritis, IQR = interquartile range, FIHOA = Functional Index Hand OsteoArthritis, KL = Kellgren Lawrence, KOOS = Knee Injury and Osteoarthritis Outcome Score, n = number, SD = standard deviation, ˆ=according to the American College of Rheumatology classification criteria for clinical OA [17,21]. KOOS = Knee Injury and Osteoarthritis Outcome Score, NRS = Numeric Rating Scale

### 3.2 Radiographic progression according to the OARSI atlas and Kellgren-Lawrence

At baseline, structural OA (any joint with KL ​≧ ​0) was present in the hands for 154 patients (69%) and in the knees for 127 (57%). 212 patients (97%) had any hand joint with KL ​≧ ​1, and 192 patients (87%) had any knee joint with KL ​≧ ​1 (87%). Radiographic hand OA scores at baseline and year two, as well as the number of patients with progression are shown in Table 2. The minimal detectable change did not exceed two for any score. The difference between baseline and year two (delta score) for all hand and knee scores are shown in supplementary file 1. Progression (=increase larger than the minimal detectable change) of the hands was present for 66 patients (30%) for osteophytes, 41 (18%) for JSN and 56 (25%) for KL. Progression of the knees was present for 80 patients (36%) for osteophytes, 31 (14%) for JSN and 53 (24%) for KL.

**Table 2:** Structural hand and knee osteoarthritis scores (n ​= ​221).

Score (range)	Baseline (median, interquartile range)	Year two (median, interquartile range)	Minimal detectable change	Participants with progression ​> ​MDC (n, %)
**Hands**				
OP sum score (range: 0–58)	10 (5–27)	10.5 (6–19)	1.70	66 (30%)
JSN sum score (range: 0–58)	4 (1–9)	5 (1–11)	1.98	41 (18%)
KL sum score (range: 0–120)	16 (9–27)	17 (10–29)	1.73	56 (25%)
**Knees**				
OP sum score (OARSI atlas) (range: 0–24)	4 (1–7)	5 (2–9)	1.52	80 (36%)
JSN sum score (OARSI atlas) (range: 0–12)	1 (0–3)	2 (1–4)	1.24	31 (14%)
KL sum severity score (range: 0–8)	3 (1–4)	3 (2–5)	0.89	53 (24%)

Progression was defined as a change between baseline and year two larger than minimal detectable change. Abbreviations: MDC ​= ​minimal detectable change, OP = Osteophyte, JSN ​= ​joint space narrowing, KL = Kellgren and Lawrence, OARSI = OsteoArthritis Research Society International.

Comparing baseline characteristics of those with and without progression of the knees, we found that those with osteophyte progression and those with JSN progression according to the OARSI atlas had a statistically significantly higher BMI, NRS knee pain, and lower KOOS pain and function than those without progression (data not shown). Those with osteophyte progression also statistically significantly more often had structural knee OA (60/79 (76%)) than those without osteophyte progression (59/133 (44%) odds ratio (OR) 3.9 (CI 2.1–7.4)).

### 3.3 Association of hand OA severity and progression with knee OA progression according to the OARSI atlas and Kellgren-Lawrence

Osteophytes, JSN and KL of the hands at baseline were not statistically significantly associated with progression of the knees in crude analyses and after adjustment for age, sex and body mass index (Table 3).

**Table 3:** Odds ratios for hand OA severity versus knee OA progression in two years.

**Progression of osteophytes of the knees**				

	**Crude odds ratio, OARSI atlas** (95% CI)	**Adjusted odds ratio**^a^**, OARSI atlas** (95% CI)	**Crude odds ratio, KIDA** (95% CI)	**Adjusted odds ratio**^a^**, KIDA** (95% CI)
Baseline hand osteophytes severity (range 0–58)	0.99 (0.96–1.01)	0.98 (0.95–1.01)	1.03 (1.01–1.06)	1.03 (1.003–1.059)
Baseline hand JSN severity (range 0–58)	0.98 (0.95–1.01)	0.98 (0.945:1.003)	1.02 (0.997–1.05)	1.02 (0.99–1.05)
**Progression of JSN/JSW of the knees**				

	**Crude odds ratio, OARSI atlas** (95% CI)	**Adjusted odds ratio**^a^**, OARSI atlas** (95% CI)	**Crude odds ratio, KIDA** (95% CI)	**Adjusted odds ratio**^a^**, KIDA** (95% CI)
Baseline hand osteophytes severity (range 0–58)	1.03 (0.996–1.06)	1.02 (0.99–1.05)	0.99 (0.94–1.03)	0.99 (0.93–1.04)
Baseline hand JSN severity (range 0–58)	1.02 (0.98–1.05)	1.01 (0.97–1.05)	0.96 (0.89–1.01)	0.96 (0.88–1.02)
**Progression of KL scores of the knees**				

	**Crude odds ratio, OARSI atlas** (95% CI)		**Adjusted odds ratio**^a^**, OARSI atlas** (95% CI)	
Baseline KL hand severity score (range 0–120)	0.99 (0.97–1.01)		0.99 (0.97–1.01)	

The odds ratios are for structural progression of the knees for each point increase in the concerning structural hand OA score. Progression was defined as a difference between baseline and two years of follow-up larger than the minimal detectable difference for the concerning score. Abbreviations: OA = Osteoarthritis, OARSI = OsteoArthritis Research Society International, CI ​= ​confidence interval, JSN ​= ​joint space narrowing, KL = Kellgren and Lawrence.

a Adjusted ​= ​for baseline age, sex and BMI.

Osteophyte progression of the hands was negatively associated with osteophyte progression of the knees (adjusted OR 0.43, 95% confidence interval (CI) 0.22–0.83), and relative risk 0.77, 95% CI 0.63–0.94)) (Table 4). The association of JSN progression of the hands with osteophyte progression of the knees was similar (adjusted OR 0.43, 95% CI 0.18–0.93, and relative risk 0.87, 95% CI 0.77–0.98).

**Table 4:** Odds ratios for hand OA progression versus knee OA progression according to the OARSI atlas in two years.

**Progression of osteophytes of the knees**			

**Progression of the hands**	**Progression of the knees, no, n (%)**	**Progression of the knees, yes, n (%)**	**Adjusted** **Odds ratio**^a^ (95% CI)
Osteophytes, no	85 (40%)	63 (30%)	1 (reference)
Osteophytes, yes	47 (22%)	16 (8%)	0.43 (0.22–0.83)
JSN, no	102 (48%)	70 (33%)	1 (reference)
JSN, yes	30 (14%)	9 (4%)	0.43 (0.18–0.93)
**Progression of JSN of the knees**			

**Progression of the hands**	**Progression of the knees, no, n (%)**	**Progression of the knees, yes, n (%)**	**Adjusted** **Odds ratio**^a^ (95% CI)
Osteophytes, no	133 (62%)	16 (8%)	1 (reference)
Osteophytes, yes	49 (23%)	15 (7%)	2.61 (1.14–5.99)
JSN, no	151 (71%)	22 (10%)	1 (reference)
JSN, yes	31 (15%)	9 (4%)	1.93 (0.75–4.68)
**Progression of KL scores of the knees**			

**Progression of the hands**	**Progression of the knees, no, n (%)**	**Progression of the knees, yes, n (%)**	**Adjusted** **Odds ratio**^a^ (95% CI)
KL score progression, no	117 (55%)	41 (19%)	1 (reference)
KL score progression, yes	42 (20%)	12 (6%)	0.83 (0.39–1.71)

The odds ratios are for structural progression of the knees for those with hand OA progression versus no hand OA progression of the concerning feature. Progression was defined as a difference between baseline and two years of follow-up larger than the minimal detectable difference for the concerning score. Analyses in this table solely involve those with data available on the hands and knees for the concerning outcome. Abbreviations: OA = Osteoarthritis, CI ​= ​confidence interval, JSN ​= ​joint space narrowing, KL = Kellgren and Lawrence.

a Adjusted ​= ​for baseline age, sex and BMI.

JSN progression of the hands was not statistically significantly associated with JSN progression of the knees (crude and adjusted), but osteophyte progression of the hands was positively associated with JSN progression of the knees (Table 4) (adjusted OR 2.61, 95% CI 1.14–5.99), relative risk 1.42, 95% ​CI 0.99–2.01).

Since results for osteophytes and JSN progression were different, we investigated osteophyte progression and JSN progression within the hand and knee joints separately. We found that osteophyte progression of the hands was clearly associated with JSN progression of the hands (OR 9.3, 95% CI 4.3–20.1). For the knees this association between osteophyte progression and JSN progression was less obvious (OR 1.96, 95% CI 0.90–4.28).

KL score progression of the hands was not statistically significantly associated with KL score progression of the knees (adjusted OR 0.83, 95% CI 0.39–1.71), relative risk 0.86, 95% CI 0.49–1.50).

### 3.4 Association of baseline hand OA severity or progression with knee OA progression according to KIDA

KIDA data was available for 210 patients, of whom the “most affected compartment” of the index knee was the medial compartment in 185 patients (88%) and the lateral compartment in 35 patients (12%). Osteophyte progression of the most affected compartment larger than the minimal detectable change was present for 79 patients (38%). Of these patients, 22 (28%) had osteophyte progression of the hands and 13 (17%) had JSN progression of the hands. Regarding JSW, 19 patients (9%) had progression of the most affected compartment of the index knee according to KIDA, of whom three (16%) had osteophyte progression of the hands and one (5%) had JSN progression of the hands.

Baseline osteophyte severity of the hands was associated with osteophyte progression of the most affected compartment of the index knee (adjusted 1.03 (1.01–1.06)), but not with JSW progression of the most affected compartment (Table 3). Baseline JSN severity was not statistically significantly associated with osteophyte or JSW progression crude and adjusted (Table 3).

Osteophyte progression of the hands and JSN progression of the hands were not statistically significantly associated with osteophyte progression or JSW progression of the most affected compartment of the index knee (Table 5). Due to insufficient size of the group with JSN progression of the hands and osteophytes or JSW progression of the knees according to KIDA, no odds ratios for these associations were calculated.

**Table 5:** The association of hand OA severity and progression versus knee OA progression according to KIDA over two years.

**Progression of knee osteophytes, KIDA**			

**Progression of the hands**	**Progression of the knees, no, n (%)**	**Progression of the knees, yes, n (%)**	**Adjusted** **Odds ratio**^a^**(95% CI)**
Osteophytes, no	92 (44%)	57 (27%)	1 (reference)
Osteophytes, yes	38 (28%)	22 (11%)	0.83 (0.45–1.61)
JSN, no	106 (51%)	66 (32%)	1 (reference)
JSN, yes	24 (11%)	13 (6%)	0.86 (0.40–1.79)
**Progression of JSW of the knees, KIDA**			

**Progression of the hands**	**Progression of the knees, no, n (%)**	**Progression of the knees, yes, n (%)**	**Adjusted** **Odds ratio**^a^ (95% CI)
Osteophytes, no	133 (64%)	16 (8%)	1 (reference)
Osteophytes, yes	57 (27%)	3 (1%)	n/a (insufficient data)
JSW, no	154 (74%)	18 (9%)	1 (reference)
JSW, yes	36 (17%)	1 (0%)	n/a (insufficient data)

The odds ratios are for structural progression of the knees for those with hand OA progression versus no hand OA progression of the concerning feature. Progression was defined as a difference between baseline and two years of follow-up larger than the minimal detectable difference for the concerning score. Analyses in this table solely involve those with data available on the hands and knees for the concerning outcome. Abbreviations: OA = Osteoarthritis, CI ​= ​confidence interval, JSW ​= ​joint space width, KL = Kellgren and Lawrence.

a Adjusted ​= ​for baseline age, sex and BMI.

### 3.5 Comparison of progression according to the OARSI scoring method and KIDA

Of the 202 patients with knee osteophyte progression data according to the OARSI atlas and KIDA available, 36 (18%) had osteophyte progression according to both scoring systems, and 92 (46%) had no progression according to both. For JSN/JSW (204 patients), 6 (3%) had progression according to both and 168 (82%) according to neither.

### 3.6 Sensitivity analysis

The outcomes of the sensitivity analysis (exclusion of patients with OA-related knee or hand surgery) were consistent with the other analyses in this study (data not shown). Three patients had surgery during follow-up (all three for the knees), of whom none had hand OA progression of any feature (osteophytes, JSN or KL).

## 4 Discussion

We studied the association of structural hand OA and its progression with structural knee OA progression over two years. The severity of baseline radiographic abnormalities of both hands was not statistically significantly associated with radiographic progression of both knees. Osteophyte and JSN progression of the hands were positively associated with JSN progression of the knees, whereas these were negatively associated with osteophyte progression of the knees. Therefore, opposing results were found for progression of osteophytes versus progression of JSN of the knees. This difference could be due to several reasons. First, OA progresses slowly [6,14]. Therefore, the relatively short follow-up time might be inadequate for finding clear associations. Second, studies on knee OA progression are prone to methodological issues affecting study results [32]. For example, loss to follow-up (58 out of 297 patients in our study) could affect the results as well as a ceiling effect; some joints already had the maximum score at baseline for osteophytes, JSN or KL and could therefore not progress according to these outcome measures, although in reality progression might have been present. Also, investigating OA progression in a population selected on presence and even likeliness of progression of OA is prone to lead to index event bias and collider stratification bias. All of these issues can lead to a biased effect towards the null or to an inverse effect. Finally, CIs around several of the estimates, particularly the negative associations, were wide, so the occurrence of a chance finding whereas in reality there was no association, is plausible.

In our analyses using KIDA for defining knee OA progression, we could not confirm the associations we found using scores of the OARSI atlas. However, additional analyses showed that progressors according to the OARSI atlas often were not progressors according to the KIDA. Consequently, being a progressor likely depends on the method of quantification, and the OARSI atlas and KIDA might quantify different aspects of OA. These findings indicate the importance of methodological guidelines on how to define knee OA progression.

Despite the aforementioned absence of consistent associations, ORs for osteophyte progression of the knees were all lower than those for JSN progression of the knees (Table 3). Perhaps, as patients were selected based on likeliness of JSN progression of the knees, those with osteophyte progression are relatively underrepresented [16]. Another explanation is that patients with knee osteophyte progression might havea specific phenotype of OA that is not prone to hand OA progression.

The magnitude of the association between progression of the hands and knees using the KL scoring method was between that of osteophytes and of JSN. This could be explained by KL score taking into account both osteophytes and JSN [26].

We compared our outcomes with previous studies. Hassett et al. investigated 133 women with knee OA defined by presence of osteophytes for knee osteophyte progression, and 148 women with knee OA defined by presence of JSN for JSN progression over ten years of follow-up [13]. Hand osteophyte progression was not statistically significantly associated with knee osteophyte progression and not with knee JSN progression, and hand JSN progression was also not statistically significantly associated with knee osteophyte progression and not with knee JSN progression. These results are in line with our study, although study population, design and follow-up time were quite different. Felson et al. investigated 765 participants (59% women, mean age 63) [33]. No statistically significant association was found between baseline osteophytes and JSN severity of the hands versus progression of KL scores of the knees at year one of follow-up (crude and adjusted for age, sex and BMI). This is in line with our study, as baseline KL score of the hands was not associated with KL progression of the knees. Bijsterbosch et al. investigated 236 Dutch sibling pairs with symptomatic hand OA (mean age 59, 83% women) [12]. Progression was defined as an increase in either osteophytes or JSN above the minimal detectable change after six years of follow-up. Contrary to our study, hand OA progression was statistically significantly associated with knee OA progression (OR 2.3 (95% CI 1.3 to 4.0) adjusted for age, sex and BMI). This could be explained by a difference in phenotype with our study, being familial generalized OA, and difference in follow-up duration. Dahagin et al. investigated 1235 Dutch participants without definite OA of the knee (defined as KL score ≤1) (mean baseline age 65.8, 57.5% women) [11]. Participants were followed for 6.6 years. Incident knee OA was defined as any knee with KL ​≧ ​0, and structural hand OA as KL ​≧ ​0 in 2 of 3 joint groups (DIP/IP, PIP, and CMC1/STT joints) of any hand. In contrast with our study, hand OA presence at baseline was statistically significantly associated with the development of knee OA (OR 1.6 (95% CI 1.0–2.8)). This study differs from our study by including only participants with knees with a KL 0 or 1 score at baseline, and by having a follow-up time of more than six years, which might explain the differences in results with our study.

Our study has several strengths. It is the first study on the association between structural hand and knee OA using three well-established semi-quantitative scoring procedures as well as a second analysis using quantitative scoring methods. Also, the fact that our cohort comprises patients from several countries, with wide-ranging structural hand and knee OA severity enhance generalizability. However, there are some notable limitations to our study. First, there was a significant part of our study group lost to follow-up. There was inadequate follow-up data for 58/297 (20%), possibly due to the COVID-19 lockdown. However, because baseline characteristics of those lost to follow-up were mostly comparable with those included, this effect should be limited. Another limitation might be the relatively short follow-up duration. As OA generally progresses slowly, a longer follow-up duration might have led to more evident results, although the selection on likeliness of JSN knee progression should partially solve this. Another limitation might be that residual confounding is present in our associations, for example due to unmeasured factors such as physical labour and genetics. Finally, our cohort consists of patients with symptomatic knee OA, yet only 57% has structural knee OA (KL ​≧ ​0 in any knee). This could have negatively influenced the progression rate. However, it must be noted that 87% had any knee with some OA pathology (KL ​≧ ​1 in any knee). Also, 97% of our study population is of white ethnicity, which limits generalization of our results to populations of other ethnicity.

In conclusion, we found no consistent associations between baseline hand OA scores or hand OA progression with knee OA progression after two years. Combining our data with results from earlier literature, hand OA severity and progression seem not to be associated with knee OA progression in a study population not at risk for generalized OA. Whether shortcomings in methodology, or limitations in study design and duration of follow-up might play a role needs further investigation. Also, the clinical relevance of structural hand and knee OA progression needs more research.

## Author contributions

SEST contributed to the design of the study, data analysis and interpretation and drafting of the article. LS, MJ, FRR, and MK contributed to study design, data interpretation and critically revising of the article. F Berenbaum, F Blanco, IKH, SCM and HW contributed to critically revising the article, and in the acquisition of data for the work. MK was the principle investigator. All authors give final approval of the submitted article, and all authors agree to be accountable for all aspects of the work in ensuring that questions related to the accuracy or integrity of any part of the work are appropriately investigated and resolved.

## Other contributors

The authors would like to thank the clinical staff, data managers and APPROACH participants at each of the medical centers of the APPROACH study for their contributions in acquiring the clinical and imaging data.

## Role of funding source

The research leading to these results have received support from the 10.13039/501100010767Innovative Medicines Initiative Joint Undertaking under Grant Agreement no 115770, resources of which are composed of financial contribution from the European Union's Seventh Framework Programme (FP7/2007–2013) and EFPIA companies in-kind contribution. See www.imi.europa.eu and www.approachproject.eu.’

## Statement of role of funding source in publication

The financial contributors to this work had no role in the study design, collection, analysis and interpretation of data; in the writing of the manuscript; or in the decision to submit the manuscript for publication.

## Declaration of competing interest

F Berenbaum received consulting fees from Grunenthal, GSK, Eli lilly, Novartis, Pfizer and Servier, IKH received consulting fees from novartis, GSK and Grunenthal, SCM received a grant from the Dutch Arthritis Foundation, HW received financial support for the IMI-APPROACH cohort, and MK received financial support from the Dutch Arthritis Foundation, all paid to the institution. All other authors declare no competing interests.
