# Supplementary material for: Severity and progression of structural hand OA is not associated with progression of structural knee OA: The IMI-APPROACH cohort
Source: Osteoarthr Cartil Open. 2024 May 14;6(3):100487. doi: 10.1016/j.ocarto.2024.100487 (PMC11141256; doi:10.1016/j.ocarto.2024.100487)
Supplement: Multimedia component 1 [file mmc1.pdf]

Supplementary file 1: Delta scores of the hands and knees

Table 1. Distribution of radiographic hand and knee OA changes on patient level (221 patients), two years minus baseline (OARSI scoring procedure)

Delta score	-1	0	1	2	3	4	>4	Progression, n (%)
Hands								
OP sum score (range: 0-58)	0	98	58	**37**	**16**	**6**	**7**	**66 (30%)**
JSN sum score (range: 0-58)	0	126	56	**23**	**8**	**6**	**4**	**41 (18%)**
KL sum score (range: 0-120)	0	111	49	**32**	**13**	**5**	**6**	**56 (25%)**
Knees								
OP sum score (range: 0-24)	0	76	57	**40**	**29**	**4**	**7**	**80 (36%)**
JSN sum score (range: 0-12)	0	199	65	**24**	**5**	**1**	**1**	**31 (14%)**
KL sum score (range: 0-8)	1	161	**39**	**14**	**0**	**0**	**0**	**53 (24%)**

Differences larger than the minimal detectable change are bold. Abbreviations: OA = osteoarthritis, OARSI = OsteoArthritis Research Society International, OP = Osteophyte, JSN = joint space narrowing, KL = Kellgren and Lawrence.
